# The Intergenerational Transmission of Chronic Pain from Parents to Survivors of Childhood Cancer

**DOI:** 10.3390/children7110246

**Published:** 2020-11-21

**Authors:** Michaela Patton, Mehak Stokoe, Caitlin Forbes, Chidera Nwaroh, Melanie Noel, Kathleen Reynolds, Fiona Schulte

**Affiliations:** 1 Department of Psychology, University of Calgary, Calgary, AB T2N 1N4, Canada; michaela.patton@ucalgary.ca (M.P.); melanie.noel@ucalgary.ca (M.N.); 2 Hematology, Oncology, and Bone Marrow Transplant Program, Alberta Children’s Hospital, Calgary, AB T3B 6A8, Canada; Mehak.Sandhu@albertahealthservices.ca (M.S.); Caitlin.Forbes@albertahealthservices.ca (C.F.); kareynol@ucalgary.ca (K.R.); 3 Cumming School of Medicine, University of Calgary, Calgary, AB T2N 1N4, Canada; chidera.nwaroh@ucalgary.ca

**Keywords:** chronic pain, oncology, pediatric, parent, survivors of childhood cancer

## Abstract

Background: Among youth with chronic non-cancer pain, 50% have parents with chronic pain. These youth report significantly more pain interference and posttraumatic stress symptoms (PTSS), and worse health-related quality of life (HRQL) than youth whose parents do not have chronic pain. Additionally, parent chronic pain is linked to increased child anxiety and depressive symptoms. Survivors of childhood cancer (SCCs) are at risk of pain and negative psychosocial outcomes and therefore may be especially vulnerable if their parents have chronic pain. Thus, the aims of the current study were to (1) identify rates of chronic pain among parents of SCCs, (2) test group differences in psychological symptoms in parents with chronic pain versus without, and (3) test group differences in pain interference, HRQL, anxiety, depression, and PTSS in SCCs with parents with chronic pain versus without. Methods: 122 SCCs (Mean age = 15.8, SD = 4.8, 45.7% male, Mean age at diagnosis = 5.9, SD = 4.7) and their parents were recruited from across Canada to complete online questionnaires. Parents were asked if they have had pain for at least three consecutive months and completed the brief symptom inventory (BSI) as a measure of psychological symptomatology. Survivors completed the pain questionnaire, patient reported outcomes measurement information system (PROMIS)—pain interference, anxiety, and depression measures, child posttraumatic stress scale, posttraumatic stress disorder checklist for the Diagnostic and Statistical Manual of Mental Disorders, and the pediatric quality of life inventory. Results: Forty-three (39%) parents of SCCs reported having chronic pain. Of the 29 survivors who had chronic pain, 14 (48%) also had parents with chronic pain. Parents with chronic pain reported significantly higher scores on the BSI than parents without chronic pain, *F*(1, 116) = 5.07, *p* = 0.026. SCCs with parents with versus without chronic pain reported significantly higher PTSS *F*(1, 105) = 10.53, *p* = 0.002 and depressive symptoms *F*(1, 102) = 6.68, *p* = 0.011. No significant differences were found across the other variables tested. Conclusions: Findings suggest that survivors’ parents’ own pain is prevalent and is related to survivors’ increased depressive symptoms and PTSS, but not anxiety, pain interference, or HRQL. Future research should explore whether parents may benefit from psychological intervention after their child has been diagnosed with cancer and how this could improve outcomes for their child.

## 1. Introduction

One in five Canadians lives with chronic pain (i.e., pain lasting > three months) [1]. When pain persists, it can affect all aspects of a person’s life including work, school, social relationships, and caregiving. Living with unmanaged pain is associated with sleep disturbances, depression, anxiety, and diminished health-related quality of life (HRQL) [2,3,4,5]. Chronic pain is prevalent among adults and there is emerging evidence that parents can transmit risk of developing chronic pain to their children [6,7,8]. A recent review found that children whose parents have chronic pain are more likely to report greater pain complaints, experience poorer psychological outcomes such as heightened anxiety and depressive symptoms, and have poorer social abilities and self-esteem than children of parents without chronic pain [7]. A conceptual model of the intergenerational transmission of chronic pain from parents to children proposes that having a parent with chronic pain heightens a child’s risk for developing chronic pain through genetic, neurobiological, pain-specific social learning, general parenting and family health, and environmental stress pathways [8]. This model provides plausible mechanisms that contribute to vulnerabilities within the child that can ultimately lead to adverse outcomes. Among youth with chronic pain, 50% have parents with chronic pain [6,9]. These youth report significantly more pain interference and posttraumatic stress symptoms (PTSS), and worse HRQL than those whose parents do not have chronic pain [6]. Additionally, parent chronic pain is related to increased child anxiety and depressive symptoms, regardless of whether or not the child has chronic pain [9].

Pain is also a problem for individuals with a history of cancer. Over 500,000 survivors of childhood cancer (SCCs) reside in North America today and this number is growing exponentially each year [10]. Unfortunately, approximately two-thirds of SCCs will experience late- and long-term effects after their treatment has completed such as chronic pain, internalizing and externalizing mental health issues, and poorer HRQL [11,12]. In fact, recent research has shown that approximately one in four SCCs lives with chronic pain and it is related to poorer HRQL and increased anxiety symptoms and PTSS [13]. SCCs may be at increased risk for many of these poor health outcomes if their parents have chronic pain; however, this has not yet been empirically examined. Thus, the aims of the current study were to: (1) identify rates of chronic pain among parents of SCCs; (2) test group differences in psychological symptomatology in parents with chronic pain versus without; and (3) test group differences in pain interference, HRQL, depressive symptoms, anxiety symptoms, and PTSS in SCCs with parents with chronic pain versus SCCs with parents without chronic pain. We hypothesize that (1) pain will be prevalent among parents of SCCs; (2) parents with chronic pain will report significantly more severe psychological symptoms than parents without chronic pain; and (3) SCCs whose parents have chronic pain will have significantly worse psychosocial outcomes (i.e., higher pain interference, depressive and anxiety symptoms, and PTSS, and significantly worse HRQL) compared to SCCs whose parents do not have chronic pain.

## 2. Materials and Methods

### 2.1. Participants

Ethics approval for this study was obtained via the local institutional review board (HREBA.CC-17-0059). Participant inclusion criteria were: SCCs of a variety of diagnoses from across Canada; current age 8–25 years; diagnosed < 21 years of age; at least 2 years post-treatment or 5 years post-diagnosis. Exclusion criteria were: acute medical issues including major injury, illness, or surgery within the past year, diagnosed psychosis or developmental disability such as autism spectrum disorder or intellectual disability, are unable to speak and read English fluently, or do not currently live in Canada. Eligible participants were recruited from the Hematology, Oncology, and Transplant Program at the Alberta Children’s Hospital in Calgary, Alberta, Canada during their regular long-term survivor follow-up visit. Additionally, eligible participants residing within Canada were recruited from social media and other online modalities such as online patient advocacy groups. One parent of each survivor was also invited to participate.

### 2.2. Measures

Pain Questionnaire. The Pain Questionnaire [14] is a seven item questionnaire designed to assess pain frequency, location, duration, intensity, affect, and chronicity. It asses for the presence of chronic pain by asking “have you had pain for at least three months in a row?” The pain questionnaire was used to assess pain among both SCCs and parents. 

Brief Symptom Inventory (BSI). The BSI [15] is a brief psychological self-report symptom scale, and was completed by parents. The 53-item scale yields numerous subscales that represent different areas of psychological health and functioning as well as a global severity index (GSI) representing the average of all 53 items. Participants are asked how much they have been distressed by each symptom in the past 7 days where each item is scored from 0 (not at all) to 4 (extremely). The BSI has demonstrated high convergence with the Minnesota Multiphasic Personality Inventory (MMPI) with good evidence of convergent validity [15]. Internal consistency for all 53 items was 0.968.

Pediatric Quality of Life (PedsQL)—Generic Core. The PedsQL—generic core [16] module is a 23-item self-report measure of HRQL which assesses physical, social, emotional, and cognitive domains. Subscores were summed to provide a total score from 0–100. Higher scores reflect better overall HRQL. This tool has been used frequently and is well validated within pediatric oncology populations for ages 8–25 years [16,17]. Internal consistency for the child (*n* = 36), adolescent (*n* = 46), and young adult (*n* = 65) measures were 0.864, 0.930, and 0.923, respectively.

Child Posttraumatic Stress Scale (CPSS-V). The CPSS-V [18] is a 17-item self-report measure for children used to assess posttraumatic stress symptoms regarding re-experiencing, avoidance, and arousal. The original CPSS was created to reflect the DSM-IV-TR criteria for post-traumatic stress disorder (PTSD), but was recently updated to reflect the Diagnostic and Statistical Manual of Mental Disorders (DSM-5) criteria for PTSD. Items are rated on a four-point Likert scale from 0 “not at all” to 3 “five or more times a week”. This tool has been found to have excellent internal consistency and good convergent validity, concurrent validity, and discriminant validity in school-based adolescents [19]. It has also demonstrated excellent internal consistency and good test-retest reliability, convergent and discriminant validity among a sample of treatment-seeking children with trauma histories [20]. While the original authors proposed a three-factor model, confirmatory factor analysis supported the use of a one-factor model using a total symptom score. This measure has shown to be reliable and valid for children 8 years and older. The CPSS-V has been used in pediatric cancer populations [21]. The CPSS-V was completed by survivors age 8–17 years. Internal consistency for our sample (*n* = 72) was 0.959.

Posttraumatic Stress Disorder Checklist for DSM-5 (PCL-5). The PCL-5 [22] is a 20-item self-report measure that assesses PTSD symptoms experienced over the past month, and was created in line with the DSM-5 criteria for PTSD. Each item is rated on a five-point Likert scale from 0 “not at all” to 4 “extremely” and are summed together to create a continuous measure of PTSD symptoms. This tool has been found to have excellent internal consistency, good test-retest reliability, and good convergent and discriminant validity in trauma-exposed college students [22] as well as a general population of undergraduates [23]. This measure was completed by survivors aged 18–25 years. Internal consistency for our sample (*n* = 72) was 0.936.

Patient Reported Outcomes Measurement Information System (PROMIS). The PROMIS [24] Anxiety and Depression instruments (eight-item short form) were administered to screen for current symptoms of anxiety and depression. Survivors completed the self-report instrument corresponding to their age (child: age 8–17; adult: age 18+). Items (e.g., “I felt worried”) are rated on a five-point scale from 1 “never” to 5 “always”. Higher scores signify greater severity of symptoms. Pain interference was assessed using the four-item PROMIS interference scale for survivors 8–17 years of age, and the eight item PROMIS interference scale for survivors over 18 years of age [25]. The PROMIS pediatric measures have been validated for use in pediatric oncology [25]. Internal consistency for child (*n* = 73) and young adult (*n* = 73) measures of anxiety were 0.940 and 0.955, respectively. Internal consistency for child (*n* = 74) and young adult (*n* = 72) measures of pain interference were 0.902 and 0.963, respectively. Internal consistency for child (*n* = 73) and young adult (*n* = 73) measures of depression were 0.970 and 0.947, respectively.

Demographics. Demographic information from survivors was collected including current age and ethnicity. Additional demographic information was collected from parents of survivors under 18 years old including household income, mother’s education, and father’s education. While information on fathers and mothers was collected, the questionnaire did not inquire about who was completing the survey. Information was collected from survivors about their cancer history including diagnoses, treatments, and relapse. Finally, medical information from patient charts was collected including sex, diagnosis date and description, and treatment received.

### 2.3. Procedure

Eligible participants were approached during their regular long-term survivor clinic visits to assess their interest in participating. If warranted, details of the study were provided by a member of the research team. Additionally, the study advertised on social media and other online modalities to recruit survivors across Canada. Upon agreement to participate, survivors or parents were contacted via phone to undergo screening by a member of the research team. If eligible, participants were emailed unique links to complete the questionnaire package online via research electronic data capture (REDCap) [26,27]. Survivors were given access to the questionnaire only upon completion of the appropriate electronic consent forms, located at the beginning of the questionnaire. For survivors under the age of 18 years, caregivers provided consent for their child’s participation. Survivors under the age of 18 years also provided assent. Survivors over the age of 18 years provided their own consent.

### 2.4. Statistical Analyses

To address our primary aim, descriptive statistics and frequencies were used to identify rates of chronic pain among parents of SCCs and to describe demographic variables. To address our second and third aims, one-way analysis of variance (ANOVA) was used to test group differences in BSI scores for parents with versus without chronic pain and one-way analysis of covariance (ANCOVA) was used to test group differences between survivors whose parents have chronic pain versus those who do not in psychosocial outcomes.

## 3. Results

### 3.1. Demographic Information

**Figure 1:**
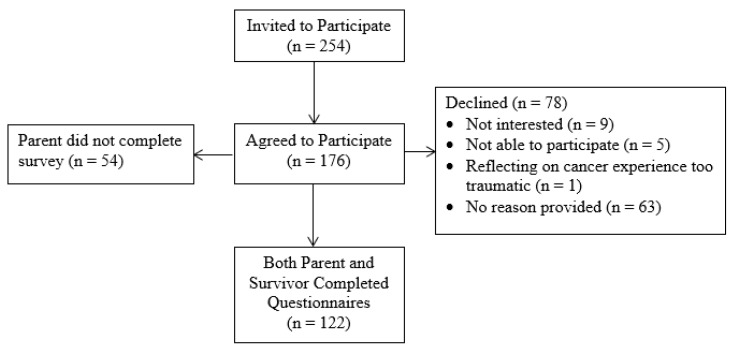
Recruitment flow chart.

Participants were 122 parent-survivor dyads. Of the 254 eligible survivors invited to participate, 176 agreed to participate, 78 declined, 54 of the survivors’ parents had not completed the survey, and 122 dyads of both survivors and parents completed the study. Reasons for declining included, “not interested” (*n* = 9), “not able to participate at this time” (*n* = 5), “reflecting on cancer experience is too traumatic” (*n* = 1), or no reason provided (*n* = 63). Recruitment and retention numbers can be found in Figure 1.

On average, survivors were 15.8 (SD = 4.8) years old, 5.9 (SD = 4.7) years at the time of diagnosis, and 45.7% male. Age of both fathers and mothers were collected, where mothers were 46.7 (SD = 6.9) years and fathers were 48.6 (SD = 7.2) years of age. The majority of survivors (82.0%) and parents (91.9%) were white. Time since diagnosis ranged from 3.3 to 22.6 years, with a mean of 10.9 (SD = 4.6) years. Additional demographic and clinical information can be found in Table 1. The relationships between survivor psychosocial outcomes and demographic variables were tested to identify potential covariates including sex, ethnicity, and current age. Sex differences were identified in HRQL (*p* < 0.001), pain interference (*p* = 0.002), anxiety symptoms (*p* = 0.043), and depressive symptoms, (*p* = 0.040), but not PTSS (*p* = 0.184). Current age was not significantly related to any psychosocial variables. Because the sample was primarily white, a binary variable white/non-white was created to test the relationship between ethnicity and psychosocial variables. Ethnicity was not significantly related to any psychosocial variables. Thus, sex was used as a covariate for analyses including HRQL, pain interferences, anxiety, and depression.

### 3.2. Aim 1: Identify Rates of Chronic Pain among Parents of SCCs

**Figure 2:**
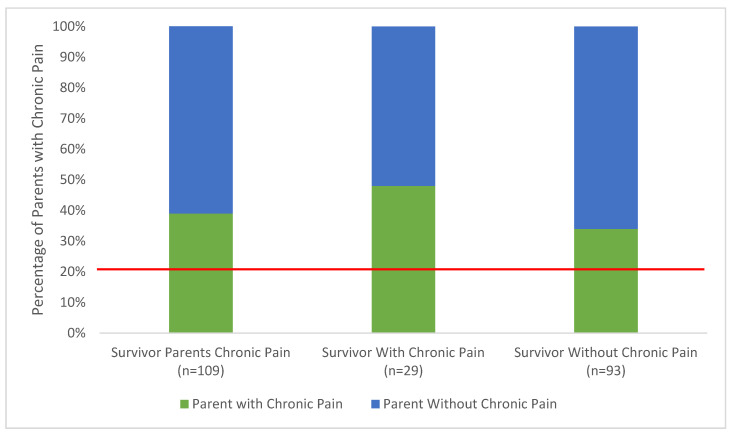
Percentage of parents of survivors of childhood cancer with chronic pain compared to the general adult population in Canada [1] represented in red.

Frequencies were used to identify rates of chronic pain in SCCs and their parents. Parents of SCCs were asked if they experienced pain that lasted at least three months, according to the definition of chronic pain [28], and 43 (39%) answered ‘yes.’ When compared to published population norms, where 20% of adults are reported to experience chronic pain, survivor parents reported significantly more chronic pain χ^2^ = 23.90, *p* < 0.000. Of the 29 survivors who reported ‘yes’ to this item, 14 (48%) also had parents with chronic pain. Parents who answered ‘yes’ to having chronic pain were also asked how long their pain has persisted, which ranged from 0.5 to 40 years with a mean duration of 9.3 (SD = 10.8) years. Findings can be found in Figure 2.

### 3.3. Aim 2: Test Group Differences in Psychological Symptoms in Parents with Chronic Pain Versus without Chronic Pain

One-way ANOVA was used to compare parents with chronic pain versus without in severity of psychological symptoms. Parents with chronic pain (Mean = 0.51, SD = 0.47) reported significantly more severe psychological symptoms than parents without chronic pain (Mean = 0.32, SD = 0.41), *F*(1, 116) = 5.07, *p* = 0.026.

### 3.4. Aim 3: Test Group Differences in Psychosocial Outcomes (i.e., Pain Interference, Quality of Life, Depression, Anxiety, and PTSS) in SCCs with Parents with Chronic Pain Versus SCCs with Parents without Chronic Pain

One-way analysis of covariance (ANCOVA) was used to compare survivors whose parents did have chronic pain versus survivors whose parents did not have chronic pain in anxiety symptoms, depressive symptoms, pain interference, and HRQL, controlling for sex. One-way analysis of variance (ANOVA) was used to compare survivors whose parents did have chronic pain versus survivors whose parents did not have chronic pain in PTSS. Survivors whose parents had chronic pain had higher depressive symptoms *F*(1, 102) = 6.68, *p* = 0.011, and PTSS *F*(1, 105) = 10.53, *p* = 0.002 compared to survivors whose parents did not have chronic pain, using a stringent alpha level of *p* < 0.01 to control for multiple comparisons. Survivors whose parents did versus did not have chronic pain did not significantly differ on scores of anxiety symptoms *F*(1, 92) = 2.24, *p* = 0.138, HRQL *F*(1, 102) = 0.77, *p* = 0.382, or pain interference *F*(1, 102) = 0.012, *p* = 0.914. Means across groups can be found in Table 2.

## 4. Discussion

The aims of the current study were to identify rates of chronic pain among parents of SCCs, test group differences in psychological symptoms in parents with versus without chronic pain, and test group differences in pain interference, HRQL, depressive symptoms, anxiety symptoms, and PTSS in SCCs with parents with chronic pain versus SCCs with parents without chronic pain. The current study found that chronic pain is prevalent among parents of SCCs and significantly greater than the general Canadian population, which suggests that one in five Canadians lives with chronic pain [1]. There are several potential hypotheses for this relationship. First, we know that parents of survivors of cancer are at risk of experiencing increased PTSS [29] which may be contributing to increased rates of chronic pain. In the chronic non-cancer pain literature, higher rates of PTSS has been linked to increased chronic pain, where youth with chronic pain have reported significantly higher PTSS compared to youth without PTSS [30]. While little research has been conducted in youth, adult samples suggest that anxiety sensitivity, avoidant coping, depression, and cognitive biases such as attentional biases towards threat underlie the co-occurrence of chronic pain and posttraumatic stress disorder in adults [31]. In the case of the current study, parents with chronic pain may potentially be at increased risk of experiencing increased psychological symptomatology. Specific to this population, parents with chronic pain could be more vulnerable to experiencing increased psychological symptoms during their child’s cancer treatment compared to parents who do not have chronic pain. The increased distress experienced during treatment may worsen their own pain and make them less equipped to support their child during this stressful process, giving the child less opportunity to properly process the distressing experience of coping with a life-threatening illness and undergoing cancer treatment. Importantly, the mean length of chronic pain reported by parents was less than the mean time since diagnosis, suggesting the pain symptoms worsened or emerged following their child’s cancer diagnosis. No studies to date have examined chronic pain in parents of SCCs and how it may affect survivors’ commonly-reported psychological late-effects of treatment. A recent systematic review identified low-quality research on pain in SCCs [32], and thus, exploration into the intergenerational experience of pain in SCCs and their parents is a novel and important investigation. 

Importantly, almost half of SCCs with chronic pain also had parents with chronic pain, which is consistent with past literature [6,9]. According to the proposed model of the intergenerational transmission of chronic pain risk [8], many factors could potentially be at play. First, genetic components may play an important role in the development of chronic pain, which could explain why there is a high co-occurrence of parent and survivor chronic pain. Early neurobiological development may be affected by genetic components or other environmental factors (i.e., low family cohesion, marital conflict) shared by the survivor and their parent. Moreover, survivors may learn specific behaviors from their parents who have chronic pain in order to inform their own choices in coping behaviors. Parenting strategies may also be affected by parental mental health in that poor mental health due to chronic pain may lead to pain overprotecting strategies and decreased ability to help their child cope with being diagnosed with cancer and/or their own chronic pain. Finally, mutual exposure to stressful environments could potentially trigger the onset of chronic pain. Survivors who had parents with chronic pain had significantly higher rates of PTSS. This finding aligns with research emerging on the co-occurrence of PTSS and pediatric pain [30]. Proposed models suggest that parent factors, including parental distress, cognitive biases, and protective responses to child pain, may contribute to the development and maintenance of comorbid pain and PTSS in children. Maternal overprotectiveness has been linked to increased child disability in youth with chronic pain [33]. Additionally, parental chronic pain status likely plays a critical role in the co-occurrence of chronic pain and PTSS in children, especially given the high prevalence of chronic pain in parents of SCCs.

The relationship between survivors’ pain and psychosocial outcomes is complicated as evidenced by the chronic non-cancer pain literature [34]. Specifically, it may not be the presence or absence of chronic pain in parents that has an effect on survivors’ psychosocial outcomes, but instead how the presence or absence of chronic pain may affect parenting, or instead, parent mental health that then plays a role in survivor functioning. One study found that mothers with chronic pain were more likely to engage in lax parenting, which was related to reduced parent-child relationship quality, and over-reactive parenting mediated the relationship between the mother’s physical functioning and child adjustment [35]. These studies demonstrate how chronic pain affects parent functioning, parenting, and mental health which, in turn, affects their child’s psychological and functional outcomes. How the experience of pain may effect parenting among SCCs is an area worthy of future exploration. 

## 5. Implications for Pain Management

This research has important clinical implications. SCCs whose parents have chronic pain may be at greater risk of developing chronic pain themselves and could also be at greater risk of experiencing psychological symptomatology. Furthermore, parents of SCCs who have chronic pain may be less equipped to meet survivors’ needs and might require additional support. As such, identifying parents of children undergoing cancer treatment who have chronic pain is an essential first step in providing parents these necessary supports. Once identified, parents with chronic pain may benefit from additional psychosocial care in order to manage their distress so that they are better equipped to meet their child’s needs. This care may entail something as simple as having a comfortable space to sit or lie down while their child is in the hospital, so as not to exacerbate their pain. Finally, parents of survivors may also benefit from pain management interventions for themselves which may, in turn, improve survivors’ outcomes. This family systems approach to understanding late effects in SCCs has been well documented in the pediatric oncology literature [36,37,38].

Psychological interventions have shown promise in helping individuals manage their pain as well as aid in our understanding of factors that influence pain and pain management. For example, mindfulness meditation has demonstrated some evidence for the reduction of pain itself as well as the ability to reduce symptoms of depression and improve HRQL [39]. Cognitive behavioral therapy has also shown promise in improving pain, disability, mood, and catastrophizing thoughts around pain [40]. Parents may also benefit from therapies targeting their psychological symptomatology. As a preventive measure, better emotional support after their child’s cancer diagnosis may help bolster their coping skills and help minimize distress. Finally, it has been proposed that many mutual factors work to maintain distress and chronic pain that could be targeted in cognitive therapy including attentional and reasoning biases, anxiety sensitivity, reminders of trauma, avoidance, depression and reduced activity levels, anxiety and pain perception, and cognitive demand from symptoms limiting the use of adaptive strategies [41]. Using this approach may help parents reduce the burden of pain and psychological distress, while also helping them learn and model more adaptive coping strategies for their child.

## 6. Limitations and Future Directions

The study was not without limitations. First, the study design was cross-sectional, which hinders our ability to draw causal conclusions or infer directionality of relationships. Longitudinal studies would aid in our understanding of the timeline in which chronic pain and psychological symptomatology unfolds as it relates to the cancer journey, for both patients and their caregivers. Additionally, larger epidemiological studies are needed to obtain larger samples of SCCs with chronic pain in order to test psychosocial differences between SCCs with chronic pain who do and do not have parents with chronic pain. Another limitation is that only one parent was assessed in the study. It is possible that parents with chronic pain were less likely to participate. In this case, the rate of chronic pain reported among parents may be an underrepresentation of the true rate in the population. An important future direction would be to assess all parents involved in each survivors’ life to obtain a better estimate of chronic pain, as well as allow for the opportunity to assess paternal versus maternal transmission of chronic pain. Due to the nature of the questionnaire, we were not able to determine if the mother or father completed the questionnaire. Additionally, the sample of parents with chronic pain was small and so larger samples would allow for sufficiently-powered analyses involving parental chronic pain status. The sample also lacked racial and ethnic diversity in that the majority of the participants self-identified as white, impeding understanding of cultural differences in the intergenerational transmission of pain. It would also be advantageous to explore mechanisms that explain why parental chronic pain is related to poor outcomes in survivors. For example, more comprehensive assessment of parent pain cognitions and behaviors and how they relate to survivors’ pain cognitions and behaviors could further explain the link between parent and child chronic pain in this population. Parental psychological factors may also play an important role in the development of functional outcomes for survivors in that parents’ own mental health as well as their tendency to catastrophize about their child’s pain may cause and maintain survivors’ disability due to pain. Future research should test models assessing the interrelationships of parent posstraumatic stress, pain catastrophizing, and survivor pain outcomes, similar to models tested in the chronic non-cancer pain literature [42]. Furthermore, pain management intervention studies for SCCs that involve parents with chronic pain are needed to test the effect of parent factors on survivors’ outcomes.

## 7. Conclusions

In sum, the current study represents the first investigation of chronic pain in parents of SCCs and thus fills an important gap in the literature. Chronic pain is known to affect caregiving and so it is vital to understand how chronic pain may affect a parents’ ability to support their child through cancer diagnosis, treatment, and beyond. Findings suggest that chronic pain is prevalent among parents of SCCs and potentially has negative consequences for both parents and survivors. Intervening at the parent level could potentially be advantageous in long-term follow-up care for SCCs.

## Figures and Tables

**Table 1:** Demographic and clinical information for survivors and their parents.

	Survivors (*n* = 122)	Parents (*n* = 122)
Current age ^†^	15.8 (4.8)	-
Sex ^‡^		
Male	48 (45.7)	-
Female	57 (54.3)	-
Age at diagnosis ^†^	5.9 (4.7)	-
Time since diagnosis (years) ^†^	10.9 (4.6)	-
Duration of chronic pain (years) ^†^	-	9.3 (10.8)
Diagnosis ^‡^		
Leukemia	37 (36.3)	-
Solid tumor	41 (40.2)	-
CNS tumor	10 (9.8)	-
Lymphoma	14 (13.7)	-
Time off treatment ^†^	9.3 (4.5)	-
Treatment ^‡^		
Chemotherapy	102 (99.0)	-
Radiation	34 (33.0)	-
Surgery	66 (64.1)	-
Transplant	15 (16.0)	-
Ethnicity ^‡^		
Caucasian	91 (82.0)	102 (91.9)
African-Canadian	2 (1.8)	0 (0.0)
East Asian	2 (1.8)	2 (1.8)
Southeast Asian	1 (0.9)	1 (0.9)
First Nations/Métis/Inuit	1 (0.9)	2 (1.8)
South Asian	1 (0.9)	2 (1.8)
Arab	3 (2.7)	2 (1.8)
Latin American	1 (0.9)	2 (1.8)
Other/Mixed Race	9 (8.1)	0 (0.0)

^†^ Mean (SD). ^‡^ N (%).

**Table 2:** Group differences in pain interference, quality of life, depression, anxiety, and posttraumatic stress symptoms (PTSS) in survivors of childhood cancer (SCCs) with parents with chronic pain versus SCCs with parents without chronic pain.

	All Survivors (*n* = 122)	Survivors of Parents with Chronic Pain (*n* = 29)	Survivors of Parents without Chronic Pain (*n* = 93)	*p*	Partial *η*^2^
Depressive symptoms ^†^	15.60 (8.29)	17.40 (10.04)	13.67 (5.55)	0.011	0.061
Anxiety symptoms ^†^	16.23 (7.81)	16.72 (6.63)	14.39 (6.25)	0.138	0.036
HRQL ^†^	75.35 (15.72)	73.06 (16.03)	76.96 (14.62)	0.382	0.007
Pain interference *,^†^	0.00 (1.00)	0.02 (1.02)	−0.03 (1.04)	0.914	0.000
PTSS **^,†^	0.00 (1.00)	0.26 (1.09)	−0.28 (0.62)	0.002	0.091 ^††^

^†^ Mean (Standard Deviation), * Pain interference was collected with the four-item patient reported outcomes measurement information system (PROMIS) measure (scale of 4–20) for survivors <18 years and eight-item PROMIS measure (scale of 8–40) for survivors ≥ 18 years. Standardized scores were used to combine the data for the whole sample. ** PTSS was measured using the child posttraumatic stress scale (CPSS) for survivors < 18 years and the posttraumatic stress disorder checklist for DSM-5 (PCL-5) for survivors ≥ 18 years. Scores were standardized to allow for combination of the data collected from both measures. ^††^ Value represents *η^2^*.
